# Supplementary material for: Thermostability profiling of MHC-bound peptides: a new dimension in immunopeptidomics and aid for immunotherapy design
Source: Nat Commun. 2020 Dec 9;11:6305. doi: 10.1038/s41467-020-20166-4 (PMC7726561; doi:10.1038/s41467-020-20166-4)
Supplement: Supplementary file 1 — Supplementary Information [file 41467_2020_20166_MOESM1_ESM.pdf]

Thermostability profiling of MHC-bound peptides: a new dimension in immunopeptidomics and aid for immunotherapy design

Jappe et al.

Supplementary Information

Supplementary Fig. 1 FACS gating strategy and analysis of C1R-A*02:01 and C1R-B*07:02 cell lines. High cell surface expression of HLA-A*02:01 (**a**) or HLA-B*07:02 (**b**) was confirmed by flow cytometry after staining with W6/32 (pan HLA class I-specific monoclonal antibody), and Goat F(ab')2 Anti-Mouse IgG(H + L), Human ads-PE (1:500). The controls are unstained cells (US) and cells stained with the secondary antibody only (2O). The gating strategies for C1R-A*02:01 or C1R-B*07:02 cell lines are the same. Here, a secondary only and positive stain are illustrated for C1R-A*02:01 to show the difference for PE (**c**).

a)

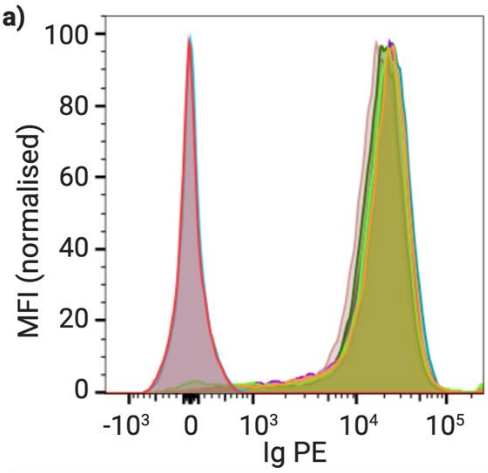

	Sample Name	Count	Mean : Comp-YG585-A
	__C1R A0201 US_001.fcs	22861	12.3
	__C1R A0201 2O_002.fcs	21510	76.1
	__C1R A0201 W632 A_003.fcs	21877	23782
	__C1R A0201 W632 B_004.fcs	22680	22719
	__C1R A0201 W632 C_005.fcs	21585	20813
	__C1R A0201 W632 D_006.fcs	21966	17674
	__C1R A0201 W632 E_007.fcs	21945	22621
	__C1R A0201 W632 F_008.fcs	21756	25566

b)

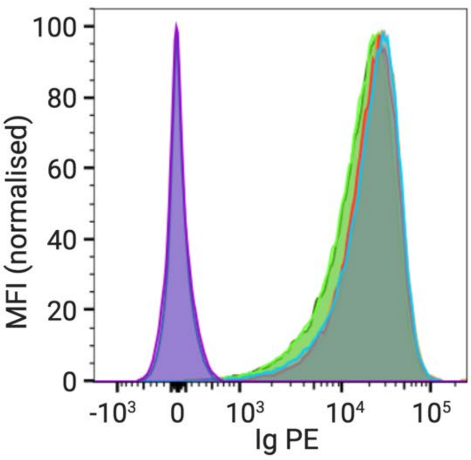

	Sample Name	Count	Mean : Comp-YG585-A
	__C1R B0702 US_001.fcs	51421	17.7
	__C1R B0702 20_002.fcs	40110	34.6
	__C1R B0702 W632 A_003.fcs	51332	25806
	__C1R B0702 W632 B_004.fcs	51082	25205
	__C1R B0702 W632 C_005.fcs	51181	26062
	__C1R B0702 W632 D_006.fcs	51405	21304
	__C1R B0702 W632 E_007.fcs	51551	21318
	__C1R B0702 W632 F_008.fcs	51289	25426

c)

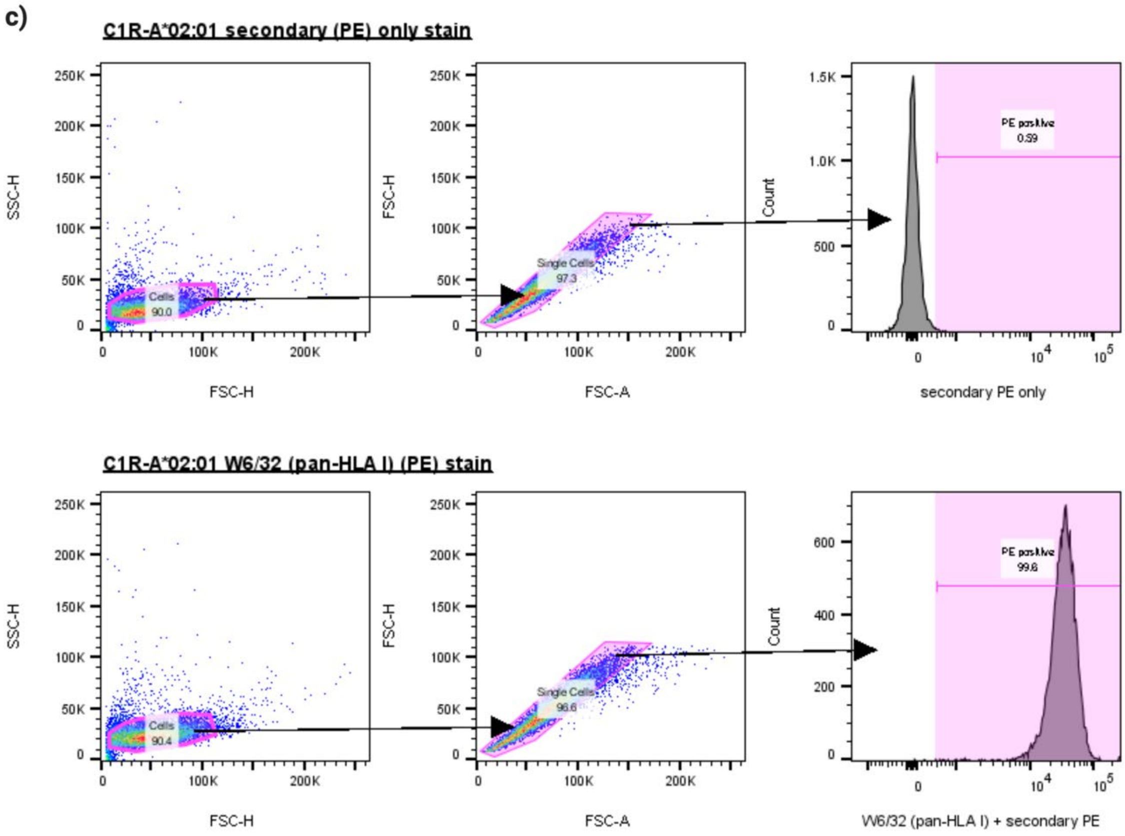

Supplementary Fig. 2 Incubation time and temperature range optimisation for the thermostability assay. a Number of peptides remaining at the reference temperature, 37°C, after a 5 min and 10 min incubation, respectively. **b** Determining the temperature at which all peptide recovery is ablated at the two selected incubation time periods. Temperature points tested are 37°C, 60°C, 70°C and 80°C.

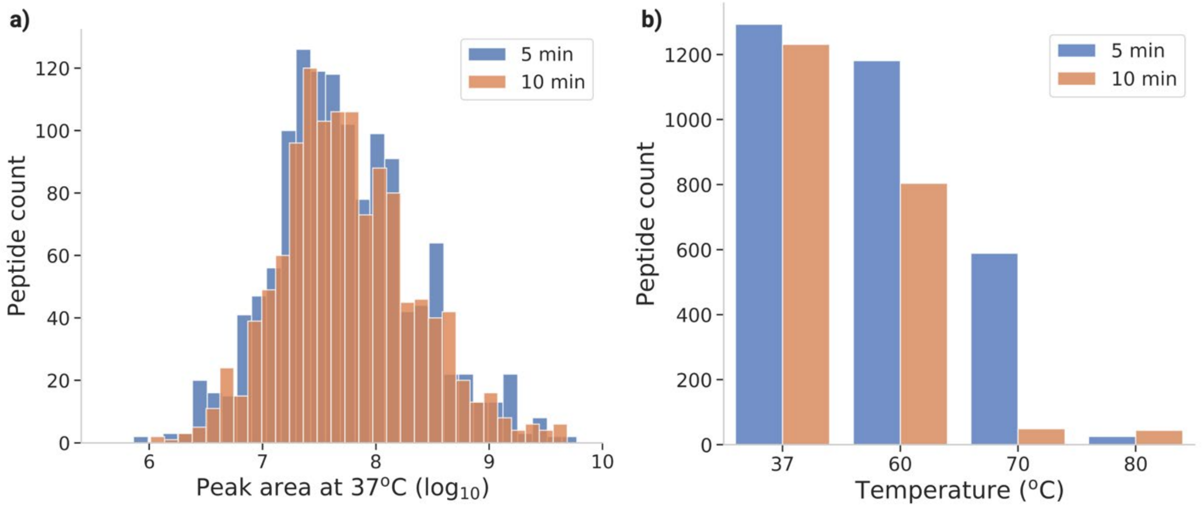

Supplementary Fig. 3 Examples of thermal melt curves for HLA-A*02:01 (a), HLA-B*07:02 (b) and HLA-C*04:01 (c). Thermal treatment of cell lysates was carried out at 12 different temperature points ranging from 37-73°C with n=3 biological replicates at each temperature point. Data are presented as median values +/- SD. Source data are provided as a Source Data file.

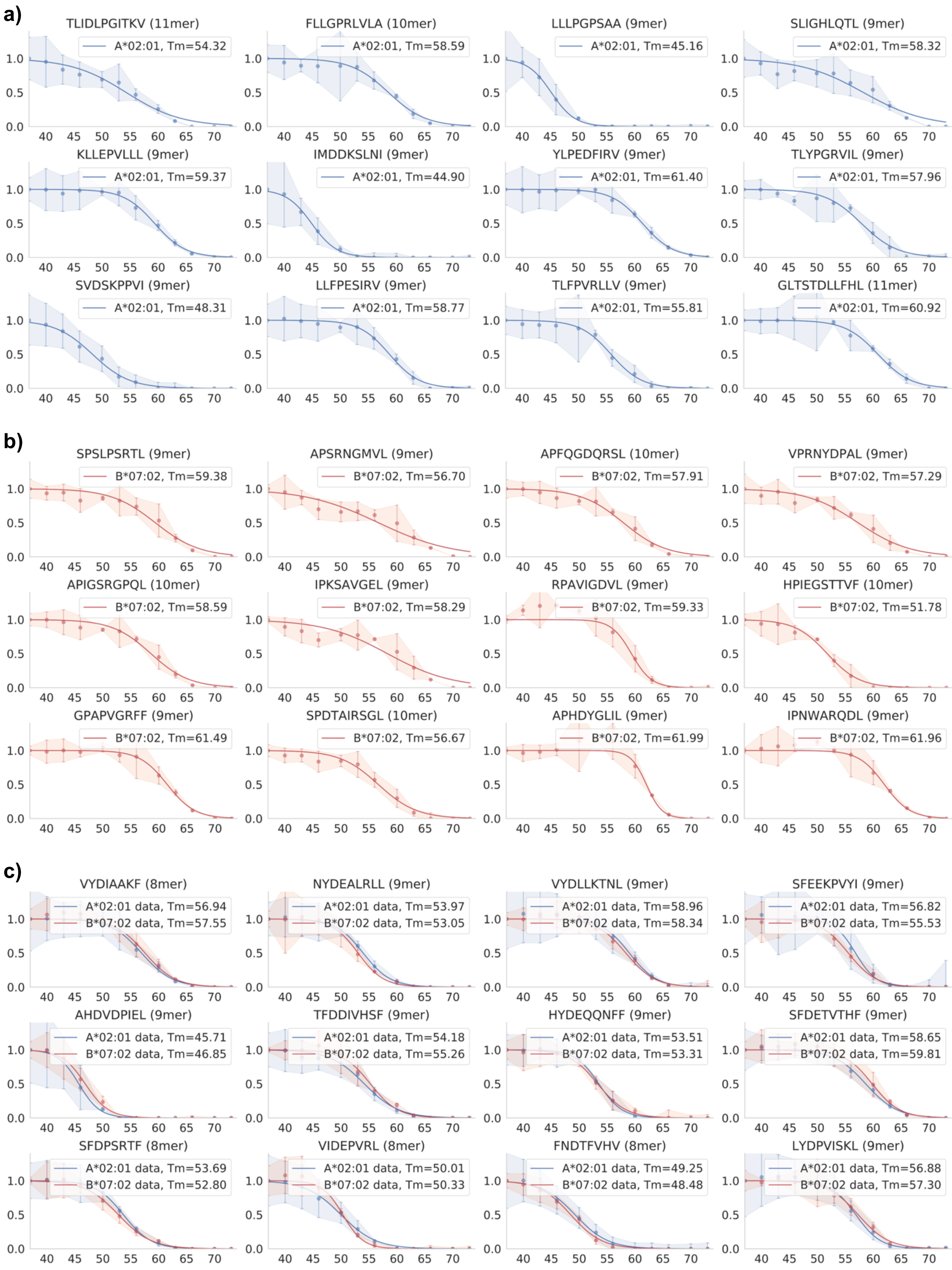

Supplementary Fig. 4 Correlation between thermostability, T_m, and half-life, $t_{½}$, of pMHC complexes. A good correlation was observed between T_m and $t_{½}$ for HLA-A*02:01 peptides identified in both the time course and thermostability assay (SCC = 0.79). Only peptides with fits satisfying $R^2>0.85$ were included in both the thermostability and kinetic stability assay. This demonstrates that thermostability provides a surrogate for kinetic stability. SCC: Spearman correlation coefficient. Source data are provided as a Source Data file.

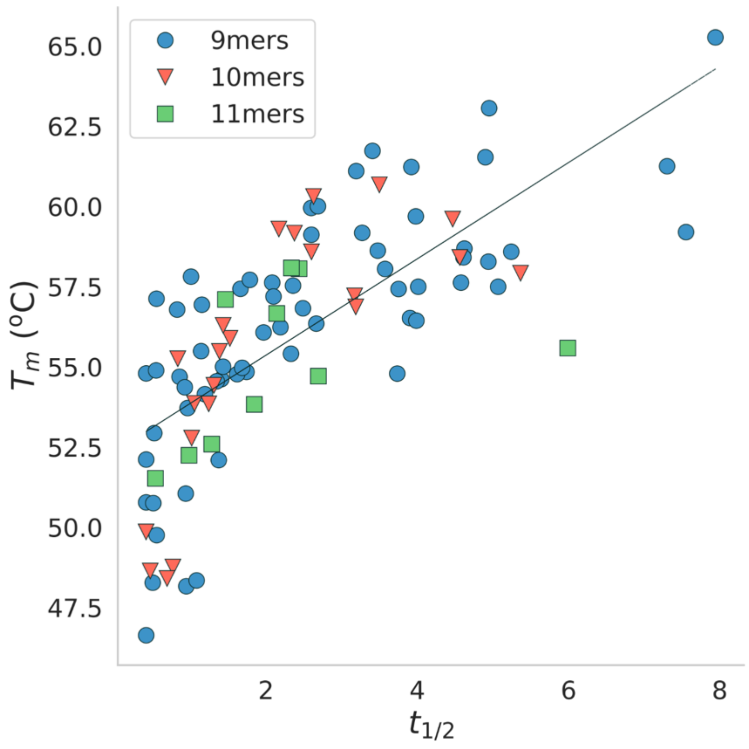

Supplementary Fig. 5 The correlation between T_m and peak intensity at 37°C is minimal. T_m values for identified peptides binding either HLA-A*02:01 (1094 peptides) or HLA-B*07:02 (1354 peptides) were visualised as a function of the median MS peak areas of the 37°C triplicates. Minimal correlation was observed for both HLA-A*02:01 (**a**) peptides (PCC = 0.10) and HLA-B*07:02 (**b**) peptides (PCC = 0.41). This demonstrated that T_m values are not an artefact of peptides ionizing with different efficiency but rather an actual representation of the stability of the pMHC complex. PCC: Pearson correlation coefficient. Source data are provided as a Source Data file.

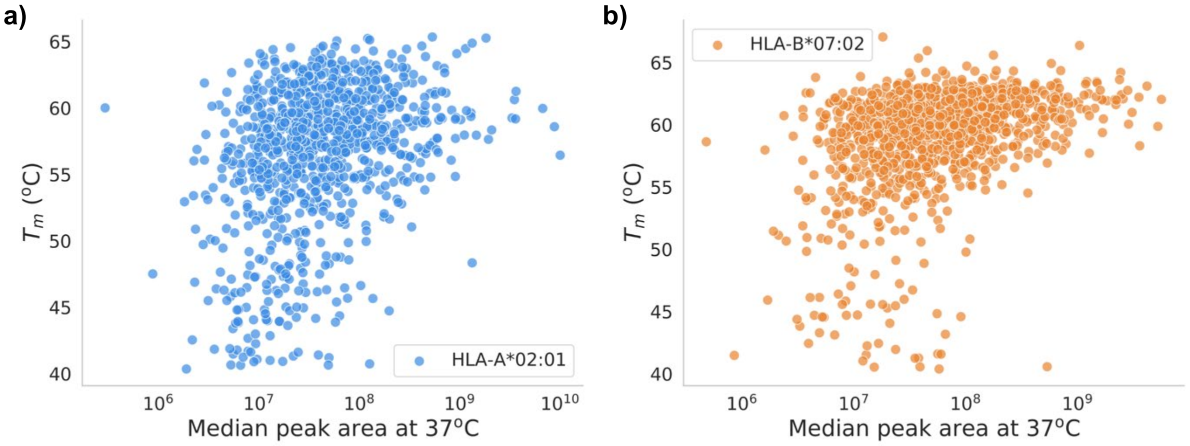

Supplementary Fig. 6 Sequence motifs for 1,500 peptides predicted to be high and low stability binders and 1,500 peptides predicted to have high and low likelihood of being an eluted ligand (EL). All binding sequence motifs were generated based on 1,500 peptide sequences predicted from a pool of filtered eluted ligands, consisting of 8,138 and 8,134 sequences for HLA-A*02:01 (**a**) and B*07:02 (**b**), respectively. The high and low stability peptides were predicted using the developed ANN-based prediction models for HLA-A*02:01 and HLA-B*07:02. These sequence motifs were compared to those generated based on peptide sequences predicted to have a high and low likelihood of being an eluted ligand according to netMHCpan-4.0[1] (**a**,**b**; right panels).

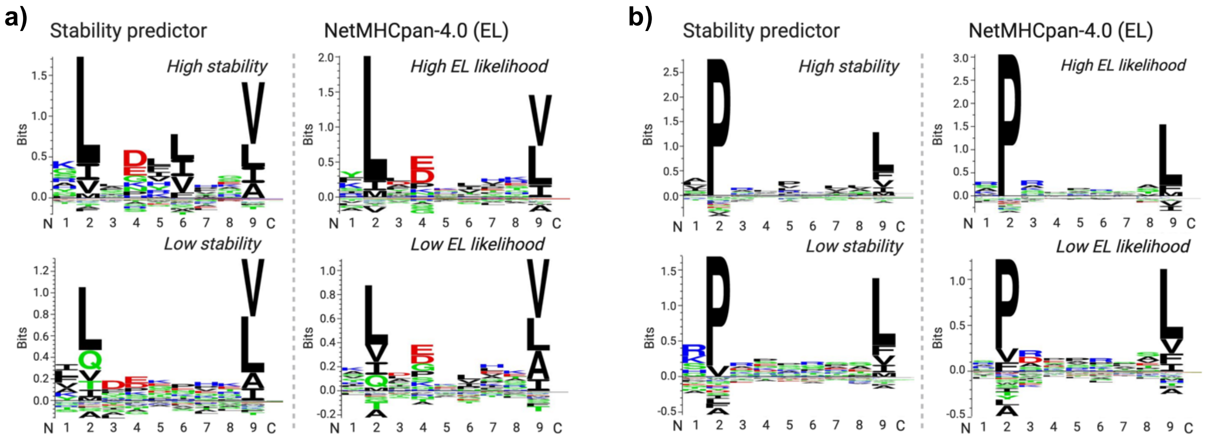

Supplementary Fig. 7 The Stability Predictor can better distinguish between immunogenic neoepitopes and non-immunogenic cancer peptides than current prediction algorithms. The prediction values of 26 immunogenic neoepitopes[2] (indicated as 'Positive' in the box plot) and 20 non-immunogenic cancer peptides, retrieved from the IEDB (indicated as 'Negative' in the box plot), were compared for the Stability Predictor and the other prediction tools included in the benchmark. The Stability Predictor is the only predictor of those included in the benchmark that enables a significant distinction ($p < 0.01$) between the prediction values of immunogenic and non-immunogenic peptides to be made; $p = 0.004$, two-sided, independent samples t test. Box plot representation of data shows the median as center, 25th percentile and 75th percentile as bounds of boxes, maximum as 75th percentile + 1.5 times the interquartile range and minimum as 25th percentile - 1.5 times the interquartile range.

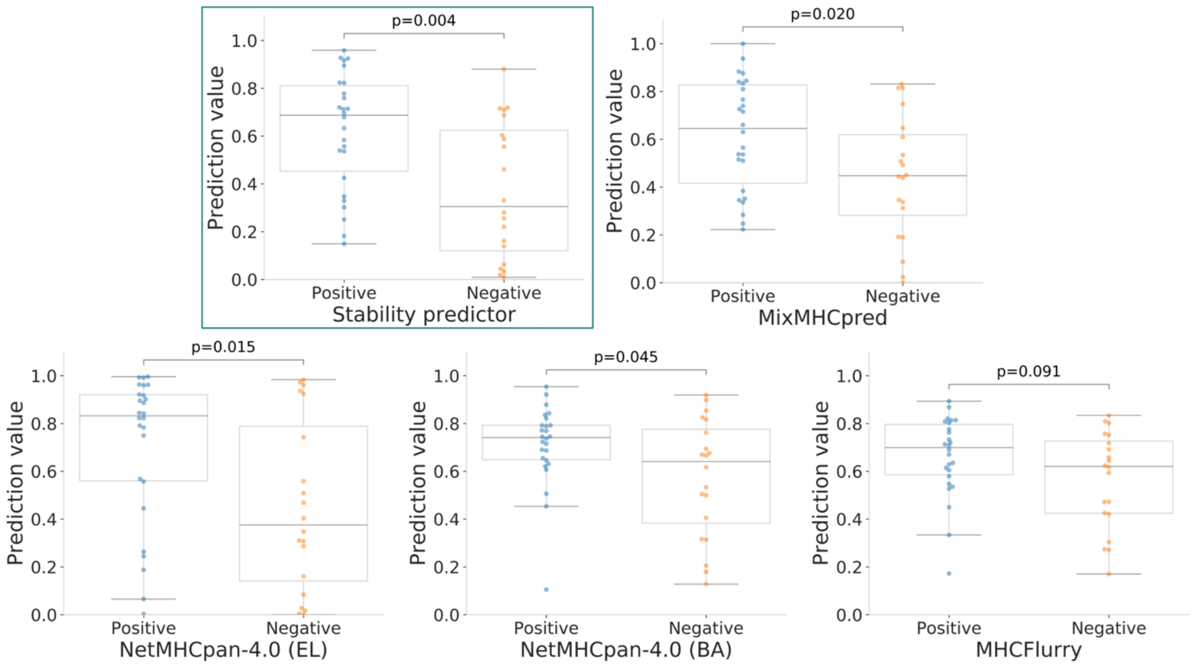

Supplementary Table 1 Outlier peptides have high predicted binding affinity to one of the other 'competing' alleles. The analysis of assay robustness using the common allele HLA-C*04:01 revealed outlier peptides that had high predicted binding affinity to one of the competing alleles, HLA-A*02:01, HLA-B*07:02 and HLA-B*35:03, expressed by the C1R-A*02:01 or C1R-B*07:02 cell lines. VLDDKLVFV and VLDDKDYFL are predicted high binders to both A*02:01 and C*04:01 and MPDDLLTTL is able to bind to both HLA-C*04:01 and HLA-B*07:02. Binding affinity was calculated using NetMHCpan-4.0 Eluted Ligand (EL) Likelihood Prediction[1] with %rank threshold for strong binders (SB) being 0.5 and weak binders (WB) being 2.0.

HLA	Peptide	%Rank	BindLevel
HLA-C*04:01	MPDDLLTTL	0.1102	<= SB
	VLDDKLVFV	0.0315	<= SB
	VLDDKDYFL	0.0317	<= SB
HLA-A*02:01	MPDDLLTTL	4.0125	
	VLDDKLVFV	0.0052	<= SB
	VLDDKDYFL	0.0112	<= SB
HLA-B*07:02	MPDDLLTTL	0.0705	<= SB
	VLDDKLVFV	10.9259	
	VLDDKDYFL	5.1535	
HLA-B*35:03	MPDDLLTTL	0.0014	<= SB
	VLDDKLVFV	1.8544	<= WB
	VLDDKDYFL	6.4081	

Supplementary Note. Time course stability study and half-life determination.

To prove that thermostability is an appropriate stability measure for pHLA, we conducted a time-course stability assay using a small-scale variation of the workflow described in the recently published protocol by Purcell *et al.*[3]. C1R-A*02:01 cells were lysed and separated into replicates equivalent of $5x10^7$ cells after which the replicates were incubated in triplicates at 37°C using a benchtop heat block for different periods of time ranging from 0-5 hrs. Following stability treatment, pHLA complexes were isolated using W6/32 antibody after which peptides were eluted and separated from the class I heavy chain, β_2m and antibody using molecular weight filters. Samples were analyzed in DIA mode, and peptides were identified by matching with the generated HLA-A*02:01 spectral library in Skyline. Samples incubated for 0 hrs represented the maximal peak area and maximal number of detected peptides and were thus used as reference to determine the relative abundance of individual pHLA. Normalized peak areas were fitted to exponential decay curves and peptides were filtered to the set with fits satisfying R^2>0.85. For the filtered peptides, half-lives ($t_{½}$) were computed using

$$t_{1/2} = \frac{\ln(2)}{\lambda} \quad \text{(S1)}$$

where λ is the decay constant. We found a good correlation between $t_{½}$ and T_m in our study (SCC=0.79) demonstrating that the latter provides a surrogate for the former (Supplementary Fig. 4).

Supplementary References

1. Jurtz, V., Paul, S. & Andreatta, M. NetMHCpan-4.0: Improved Peptide-MHC Class I Interaction Predictions Integrating Eluted Ligand and Peptide Binding Affinity Data. *J. Immunol.* **199**, 3360–3368 (2017).
2. Blaha, D. T. *et al.* High-Throughput Stability Screening of Neoantigen/HLA Complexes Improves Immunogenicity Predictions. *Cancer Immunol. Res.* **7**, 50–61 (2019).
3. Purcell, A. W., Ramarathinam, S. H. & Ternette, N. Mass spectrometry-based identification of MHC-bound peptides for immunopeptidomics. *Nat. Protoc.* **14**, 1687–1707 (2019).
