# Supplementary material for: Thermostability profiling of MHC-bound peptides: a new dimension in immunopeptidomics and aid for immunotherapy design
Source: Nat Commun. 2020 Dec 9;11:6305. doi: 10.1038/s41467-020-20166-4 (PMC7726561; doi:10.1038/s41467-020-20166-4)
Supplement: Supplementary file 3 — Descriptions of Additional Supplementary Files [file 41467_2020_20166_MOESM3_ESM.pdf]

Descriptions of Additional Supplementary Files

Supplementary Data 1

Description: List of HLA-A*02:01 ligands generated by immunoprecipitation of pHLA complexes with antibody BB7.2 using established workflows (Fig. 1a), sequencing by high-resolution mass spectrometry in DDA mode and processing in PEAKS® (Fig. 1b). Refer to enclosed excel file. Monash University Room 214, 2/F, 15 Innovation Walk, Clayton, VIC 3800, Australia +61 3 9902 9265 anthony.purcell@monash.edu https://research.monash.edu/en/persons/anthony-purcell CRICOS Provider No. 00008C ABN 12 377 614 012

Supplementary Data 2

Description: List of HLA-B*07:02 ligands generated by immunoprecipitation of pHLA complexes with antibody W6/32 using established workflows (Fig. 1a), sequencing by high-resolution mass spectrometry in DDA mode and processing in PEAKS® (Fig. 1b). Refer to enclosed excel file.

Supplementary Data 3

Description: HLA-A*02:01 ligands, their Tm values and transformed Tm values. Refer to enclosed excel file.

Supplementary Data 4

Description: HLA-B*07:02 ligands, their Tm values and transformed Tm values. Refer to enclosed excel file.

Supplementary Data 5

Description: HLA- C*04:01 ligands and their Tm values. Refer to enclosed excel file.

Supplementary Data 6

Description: Includes the list of neoepitopes (26 peptides) curated from the literature by Blaha et al., the list of cancer peptides (20 peptides) confirmed to be negative in multiple subjects tested in multimer/tetramer or ELISPOT assays, retrieved from the IEDB, and the list of all confident negatives (199 peptides) from the IEDB, including all cancer, autoimmune and viral peptides.
