# Supplementary material for: Thermostability profiling of MHC-bound peptides: a new dimension in immunopeptidomics and aid for immunotherapy design
Source: Nat Commun. 2020 Dec 9;11:6305. doi: 10.1038/s41467-020-20166-4 (PMC7726561; doi:10.1038/s41467-020-20166-4)
Supplement: Supplementary file 10 — Reporting Summary [file 41467_2020_20166_MOESM10_ESM.pdf]

nature research

Corresponding author(s): Anthony W Purcell

Last updated by author(s): Oct 28, 2020

Reporting Summary

Nature Research wishes to improve the reproducibility of the work that we publish. This form provides structure for consistency and transparency in reporting. For further information on Nature Research policies, see our Editorial Policies and the Editorial Policy Checklist.

Statistics

For all statistical analyses, confirm that the following items are present in the figure legend, table legend, main text, or Methods section.

n/a	Confirmed	
☐	☒	The exact sample size (n) for each experimental group/condition, given as a discrete number and unit of measurement
☐	☒	A statement on whether measurements were taken from distinct samples or whether the same sample was measured repeatedly
☐	☒	The statistical test(s) used AND whether they are one- or two-sided<br>*Only common tests should be described solely by name; describe more complex techniques in the Methods section.*
☐	☒	A description of all covariates tested
☐	☒	A description of any assumptions or corrections, such as tests of normality and adjustment for multiple comparisons
☐	☒	A full description of the statistical parameters including central tendency (e.g. means) or other basic estimates (e.g. regression coefficient) AND variation (e.g. standard deviation) or associated estimates of uncertainty (e.g. confidence intervals)
☐	☒	For null hypothesis testing, the test statistic (e.g. F, t, r) with confidence intervals, effect sizes, degrees of freedom and P value noted<br>*Give P values as exact values whenever suitable.*
☒	☐	For Bayesian analysis, information on the choice of priors and Markov chain Monte Carlo settings
☐	☒	For hierarchical and complex designs, identification of the appropriate level for tests and full reporting of outcomes
☐	☒	Estimates of effect sizes (e.g. Cohen's d, Pearson's r), indicating how they were calculated

Our web collection on statistics for biologists contains articles on many of the points above.

Software and code

Policy information about availability of computer code

Data collection	All LC-MS data was acquired on a Thermoscientific Q-Exactive Plus mass spectrometer using Xcaliber 3.0.63 acquisition software (ThermoScientific, San Jose, CA, USA).
Data analysis	PEAKS Studio® (v.10) (Bioinformatics Solutions Inc)<br>human UniprotKB database (v2019-08)<br>Skyline (v.4.2) (Maccoss laboratory, University of Washington, USA)<br>NNalign (Nielsen laboratory, DTU, Denmark) http://www.cbs.dtu.dk/services/NNAlign-2.0/

For manuscripts utilizing custom algorithms or software that are central to the research but not yet described in published literature, software must be made available to editors and reviewers. We strongly encourage code deposition in a community repository (e.g. GitHub). See the Nature Research guidelines for submitting code & software for further information.

Data

Policy information about availability of data

All manuscripts must include a data availability statement. This statement should provide the following information, where applicable:

- Accession codes, unique identifiers, or web links for publicly available datasets
- A list of figures that have associated raw data
- A description of any restrictions on data availability

All data are available as Supplementary Data files or available from the corresponding authors upon reasonable request. The original mass spectrometry proteomics data, the PEAKS Studio® search results and the Skyline Report files have been deposited to the ProteomeXchange Consortium via the PRIDE partner repository with the dataset identifiers PXD017824 (C1R-A*02:01 and C1R-B*07:02 DDA LC-MS/MS) and PXD017839 (C1R-A*02:01 and C1R-B*07:02 DIA LC-MS/MS for thermal stability experiments and the experiments used to determine complete ablation of peptide recovery at high temperature).

Field-specific reporting

Please select the one below that is the best fit for your research. If you are not sure, read the appropriate sections before making your selection.

☒ Life sciences ☐ Behavioural & social sciences ☐ Ecological, evolutionary & environmental sciences

For a reference copy of the document with all sections, see nature.com/documents/nr-reporting-summary-flat.pdf

Life sciences study design

All studies must disclose on these points even when the disclosure is negative.

Sample size	n/a These are large sacle data acquisiotn studies sample size is not relevant here
Data exclusions	No data was excluded from the analysis
Replication	All measurements were made in biological triplicates
Randomization	No randomization was required since the study involved direct measurement of biochemical parameters
Blinding	Blinding is not relevant to the analysis in this study

Reporting for specific materials, systems and methods

We require information from authors about some types of materials, experimental systems and methods used in many studies. Here, indicate whether each material, system or method listed is relevant to your study. If you are not sure if a list item applies to your research, read the appropriate section before selecting a response.

Materials & experimental systems

n/a	Involved in the study	
☐	☒	Antibodies
☐	☒	Eukaryotic cell lines
☒	☐	Palaeontology and archaeology
☒	☐	Animals and other organisms
☒	☐	Human research participants
☒	☐	Clinical data
☒	☐	Dual use research of concern

Methods

n/a	Involved in the study	
☒	☐	ChIP-seq
☒	☐	Flow cytometry
☒	☐	MRI-based neuroimaging

Antibodies

Antibodies used	W6/32 (produced in house from well characterized hybridoma available from ATCC), goat F(ab')2 Anti- Mouse IgG(H + L) Human adsorbed-PE (Commercially sourced from Southern Biotech and Reacts with the heavy and light chains of mouse IgG1, IgG2a, IgG2b, IgG2c, and IgG3 and with the light chains of mouse IgM and IgA), HLA-A*02:01-specific antibody BB7.2 (produced in house and available from ATCC (HB-82)).
Validation	Antibody specificity has been well documented in literature and specificty data is available on the vendors website for each batch. Each purified batch is tested by flow cytometry

Eukaryotic cell lines

Policy information about cell lines

Cell line source(s)	C1R.A2 (HLA-A*02:01 transfected C1R parental cell line), C1R.B7 (HLA-A*02:01 transfected C1R parental cell line) are both published reagents (see Faridi et al. Sci Immunol 3(28), eaar3947 (2018) and Purcell et al Nat Protocols 14, 1687–1707 (2019)).
Authentication	HLA cell surface expression was rtoutinely montired by flow cytometry using the W6/32 antibody. The parental cells have been validated by RNASeq (Faridi et al. Sci Immunol 3(28), eaar3947 (2018))
Mycoplasma contamination	All cell line were free of mycoplasma as determined by sensitive PCR.
Commonly misidentified lines (See ICLAC register)	No commonly misidentified cell lines were used in the study.
